# Sorption–Deformation–Percolation Model for Diffusion in Nanoporous Media

**DOI:** 10.1021/acsnano.2c10384

**Published:** 2023-02-27

**Authors:** Chi Zhang, Ali Shomali, Benoit Coasne, Dominique Derome, Jan Carmeliet

**Affiliations:** † Chair of Building Physics, Department of Mechanical and Process Engineering, ETH Zurich, Rämistrasse 101, 8092 Zürich, Switzerland; ‡ Université Grenoble Alpes, CNRS, LIPhy, 38000 Grenoble, France; § Department of Civil and Building Engineering, Université de Sherbrooke, Sherbrooke J1K 2R1, Québec, Canada

**Keywords:** diffusion, molecular dynamics, modulus, sorption, percolation

## Abstract

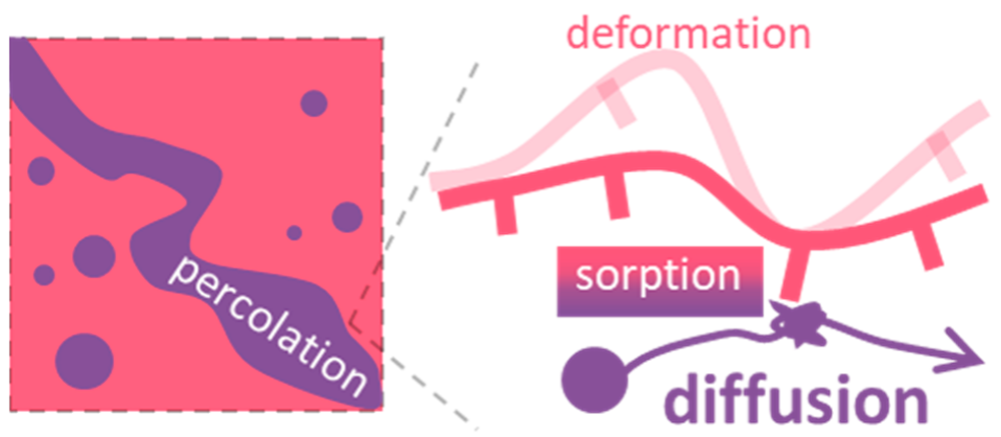

Diffusion of molecules in porous media is a critical process that is fundamental to numerous chemical, physical, and biological applications. The prevailing theoretical frameworks are challenged when explaining the complex dynamics resulting from the highly tortuous host structure and strong guest–host interactions, especially when the pore size approximates the size of diffusing molecule. This study, using molecular dynamics, formulates a semiempirical model based on theoretical considerations and factorization that offer an alternative view of diffusion and its link with the structure and behavior (sorption and deformation) of material. By analyzing the intermittent dynamics of water, microscopic self-diffusion coefficients are predicted. The apparent tortuosity, defined as the ratio of the bulk to the confined self-diffusion coefficients, is found to depend quantitatively on a limited set of material parameters: heat of adsorption, elastic modulus, and percolation probability, all of which are experimentally accessible. The proposed sorption–deformation–percolation model provides guidance on the understanding and fine-tuning of diffusion.

Diffusion of molecules, *e.g*., water, in porous media is of fundamental importance for numerous applications including drug delivery, heterogeneous catalysis, adhesives, and many others. The main measure of diffusion is the diffusion coefficient, including intrinsic, tracer, corrected, transport, mutual, self, chemical, and collective diffusion coefficients, many of which are clarified in Supplementary Note S1. Of note, this paper focuses on self-diffusion or, equivalently, tracer diffusion.^1^

Understanding how diffusion coefficient is governed by the physical parameters of porous media has led to a plethora of mechanistic models ranging from activation,^2,3^ hopping,^4^ obstruction,^5^ and free volume,^6−8^ to hydrodynamic/friction,^9,10^*etc*. For dilute polymer solutions, *e.g*., hydrogels (hydration typically higher than ∼70%), diffusion theories are relatively well established, as fluid–solid interactions can be reasonably neglected when pore size λ is much larger than the diffusant size σ, λ ≫ σ. For example, the obstruction theory successfully predicts diffusion coefficients in hydrogels by relating diffusion coefficients to the probability of finding a pore large enough to accommodate water molecules.^5^ In contrast, for the case of highly concentrated polymer solutions, where λ ∼ σ, the fluid–solid interaction force ζ becomes dominant as it roughly scales with the reciprocal of the pore length scale ζ ∼ λ^–1^ (this is due to the fact that intermolecular fluid–solid forces are integrated over the entire specific surface area, which scales as the pore surface to volume ratio). This strong fluid–solid interaction force leads to rich dynamics including molecular sieving, single-file diffusion, and anomalous diffusion.^10^ In more detail, from a thermodynamical point of view, confined water displays a complex behavior as the interaction field generated by the host medium induces dynamical heterogeneities (*e.g*., ref (11)). From a dynamical point of view, rich dynamic boundaries and nonviscous effects lead to complex multiscale dynamics (*e.g*., refs (12), (13)). A tortuous pore structure superimposes additional complexity on diffusion, whereas current diffusion theories have covered only simple pore geometries, such as cylindrical and planar pores.^14^

Besides the obstruction theory, researchers proposed free volume^6−8,15^ and activation energy-based theories.^8,16^ Molecular simulation studies have proved that free volume scales linearly with adsorbed amount.^15^ However, the experimental measurement of free volume remains sophisticated, involving positron annihilation lifetime spectroscopy,^17^ inverse gas chromatography,^18^*etc.*,^19^ combined with semiempirical interpretation. Though activation energy can be more easily measured through temperature-dependent Arrhenius processes,^20,21^ the connection between pore structure and activation energy, both relevant to diffusion, is not straightforward. Moreover, the above-mentioned theories do not consider the intermittent dynamics of the diffusant. Previous works employed tortuosity, defined as the ratio of self-diffusion coefficient of bulk water to water diffusion coefficient in pores, ξ = *D*_s,w_/*D*_μ_, to phenomenologically describe the intermittent dynamics (however, we note that this definition is not completely unambiguous, as the in-pore self-diffusivity can already differ from the bulk due to confinement effects, *i*.*e*., even in the absence of residence times at the pore surface). From a fundamental viewpoint, a microscopic explanation of tortuosity based on physical parameters is still lacking.

In short, the strong fluid–solid interaction and complex pore structure form together a nontrivial energy landscape impeding the efforts of elucidating diffusion when the size of the pore, also referred to as mesh opening or free volume, approximates the water molecular size, λ ∼ σ. To tackle these important, yet unresolved, challenges, this study resorts to molecular dynamics (MD) to address water diffusion in a dense, sorptive, and deformable polymer structure. We first segregate the microscopic diffusion coefficient from the overall diffusion coefficient through the stop-and-go formalism—which is equivalent to the concept of intermittent Brownian motion—and then rationalize the concept of tortuosity using simple material parameters involving pore structure, sorption, and deformation. Our approach provides a unifying picture of the intermittent motion of water molecules and of the quantification of complex diffusion landscape through physically well-defined material parameters. This model provides a way of predicting diffusion coefficients from adsorption heat, elastic modulus, and percolation probability.

## Results and Discussion

**Figure 1:**
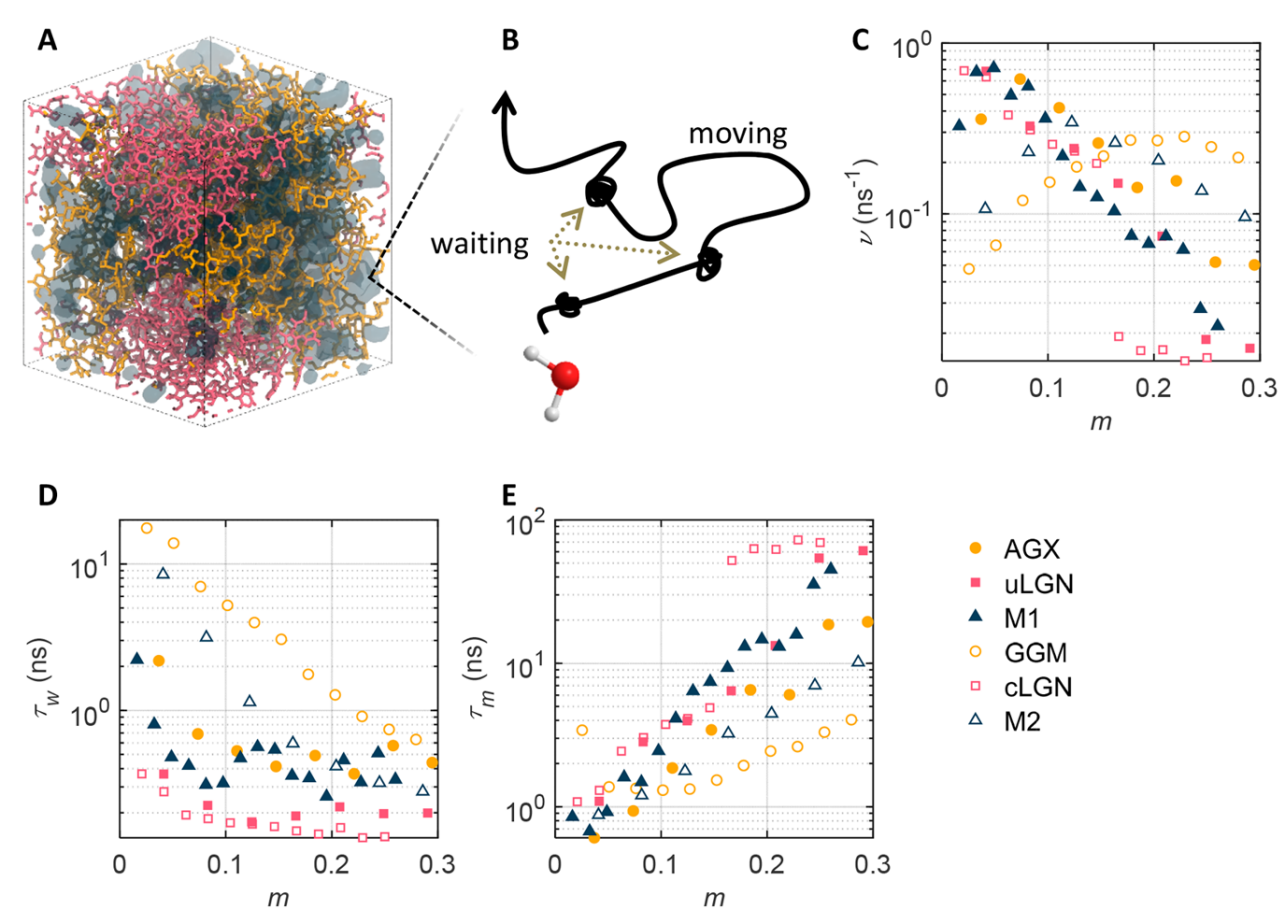
Intermittent dynamics of adsorbed water. (A) Sample system M2 at *m* ∼ 0.2. The GGM, cLGN, and water molecules are shown in yellow, pink, and dark blue colors, respectively. (B) Schematic of the intermittent motion of a single water molecule. (C) Switching frequency ν. (D) Waiting time *τ*_w_ (E) Moving time *τ*_m_ = ν^–1^ – *τ*_w_.

To study the diffusion of molecules in dense, sorptive, and deformable nanoporous structures, diffusion of water in hydrophilic nanoporous biopolymers is employed as a prototypical model. More specifically, six biopolymer systems are studied, *i*.*e*., two types of hemicelluloses, arabinoglucoronoxylan (AGX) and galactoglucomannan (GGM), two types of lignins, uncondensed lignin (uLGN) and condensed lignin (cLGN), and their mixtures, mixture 1 (M1, consisting of uLGN and AGX) and mixture 2 (M2, consisting of cLGN and GGM). These wood polymers are chosen as model systems because they cover a broad range of water–polymer interaction strengths, pore structures, chain connectivities, *etc*. More details about the molecular models are included in Supplementary Note S2 and our previous work.^22^ Moreover, water diffusion in wooden materials is relevant to ubiquitous wooden building construction, furniture, and artifacts, more specialized issues including archeological wood preservation,^23^ advanced wood-based iontronics devices,^24^*etc*. The moisture content, *m*, of the system is defined as the ratio of the mass of water to the mass of the dry polymer. This study focuses on a moisture content range *m* = 0–0.3, corresponding to the normal range of moisture load of wood exposed to atmospheric environments.^25^Figure 1a shows a sample mixture system M2 consisting of GGM and cLGN at *m* ∼ 0.2.

### Intermittent Brownian Dynamics and Diffusion Coefficients

Diffusion is usually described using Einstein’s equation where particle diffusion is considered as a random walk resulting from stochastic collisions. In sorptive systems, however, water molecules move via a series of waiting—sometimes referred to as residence/stop/immobile—and moving—relocation/go/mobile—events, alternatively identified as intermittent Brownian dynamics.^2−4,10,26,27^ Molecular motion is characterized by mean square displacement (MSD). A sample MSD curve is included in Supplementary Note S3 (Figure S1a). The MSD curve displays alternating segments of plateaus and slopes, corresponding to waiting and moving status, respectively. Figure 1b shows the schematic of the intermittent motion of a single water molecule.

Following our previous work,^26^ we define the time segment *i*, *i*.*e*., the time range *t*_*i*_ < *t* < *t*_*i*_ + 1, as waiting if the deviation from the average displacement is less than 0.1 nm (OH bond length), |***r***(*t*) – ⟨*r*⟩_*t*_ | < 0.1 nm, *t* ∈ [*t*_*i*_, *t*_*i*__+1_], and the rate of change of its squared displacement , a value based on our visual observation of water trajectories. The waiting time *t*_*i*__+1_ – *t*_*i*_ is then averaged over all waiting segments and water molecules, denoted as *τ*_w_. The frequency of status switching, *ν*, is normalized by the total simulation time *t*_tot_. Therefore, we have *t*_tot_ = *t*_w_ + *t*_m_ = τ_w_*νt*_tot_ + τ_m_*νt*_tot_, where *ν*^–1^ = τ_w_ + τ_m_ and τ_m_ is the moving time.^26^

The results of switching frequency *ν* as a function of moisture content *m* are shown in Figure 1c. For the lignins, *i*.*e*., uLGN and cLGN, the switching frequency monotonically decreases with moisture content. With the ongoing hydration process, adsorption sites at the polymer surface are gradually occupied by water molecules, and, therefore, the new water molecules in the system are gradually screened from adsorption at the occupied sites and consequently remain mostly more mobile. For the same reason, with increasing moisture content *m*, the waiting time τ_w_ decreases while the moving time τ_m_ increases as shown in Figure 1d and e. The switching frequency of the hemicellulose-containing systems, *i*.*e*., AGX, GGM, M1, and M2, displays a peak at intermediate moisture contents. This peak implies a crossover from waiting-dominated mode to moving-dominated mode, meaning that water molecules mostly stay waiting at low moisture content due to sorption and are mostly moving at high moisture content.^28^ Here, ergodicity is implicitly invoked, meaning that the number fractions of moving and waiting molecules are equal to the time fraction of moving and waiting phases, respectively. This assumption is confirmed by our simulation data.

**Figure 2:**
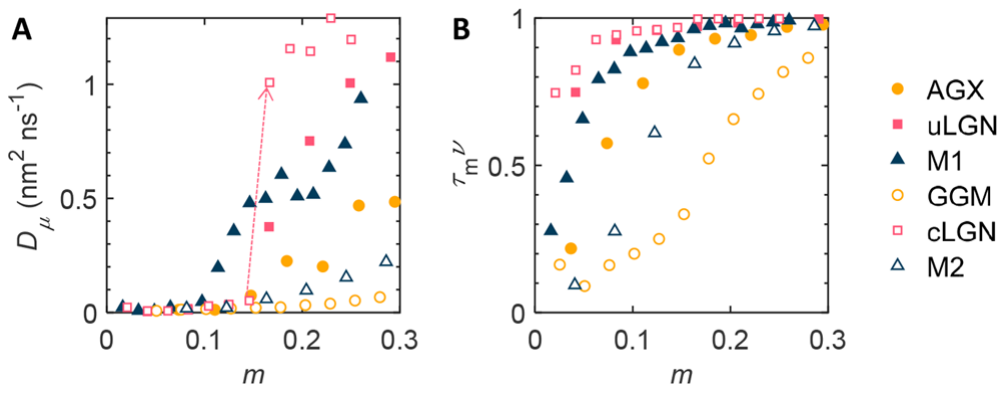
Microscopic diffusion and fraction of moving water. (A) Microscopic diffusion coefficient. The arrow indicates the surge of the diffusion coefficient of cLGN caused by percolation. (B) Fraction of moving water τ_m_*v*.

In the waiting state, water molecules are trapped, thus displaying a nearly zero diffusion coefficient. The real displacement only occurs during the moving phases (while this behavior is observed here, we note that in general surface diffusion in the adsorbed state can also be found). The diffusion coefficient in the moving phase is referred to as microscopic diffusion coefficient *D*_μ_ = *D*/(τ_m_*ν*).^26^ The factor τ_m_*ν* denotes the fraction of moving water or time with respect to the total number of water or total time. The microscopic diffusion coefficient *D*_μ_ and the fraction of moving water molecules τ_m_*v* are shown in Figure 2a and b.

The diffusion coefficient *D* is calculated as the slope of the Fickian regime of MSD averaged over all water molecules and time origins (Supplementary Note S1).^29^ For all systems, the diffusion coefficients increase with increasing moisture content but remain lower than the bulk water self-diffusion coefficient *D*_s,w_ = 3.5 nm^2^ ns^–1^ (the bulk diffusion coefficient was measured in a separate simulation). The cLGN and uLGN show the highest diffusion coefficient, while GGM the lowest. M1 shows a diffusion coefficient lower than uLGN but higher than AGX, *i*.*e*., a mixing of its two components, and so does M2. We note that the diffusion coefficient of cLGN surges at *m* ∼ 0.15 (indicated by the arrow in Figure 2a). This is attributed to a special type of water cluster structure, *i*.*e*., the formation of percolation, defined as the emergence of a water cluster that penetrates through the whole system creating a continued diffusion channel which greatly enhances diffusion coefficient. It is noted that a surge of percolation probability does not necessarily lead to a steep increase in diffusion coefficient, *e*.*g*., for GGM and AGX. There are other factors playing roles, *e*.*g*., water channel width.

According to Figure 2b, the more hydrophilic hemicelluloses, *i*.*e*., AGX and GGM, show a lower fraction of moving status. Nonetheless, at high moisture content, all systems approach nearly full moving status, *i.e.*, *τ*_m_*v* ∼ 1. This means that few water molecules remain waiting at higher moisture content. It further implies that the microscopic diffusion coefficient is actually close to the diffusion coefficient, *i.e.*, *D*_μ_ ∼ *D*. The more diffusive systems not only have a larger fraction of moving status, but also the water inside those systems moves faster, as suggested by the higher *D*_μ_.

**Figure 3:**
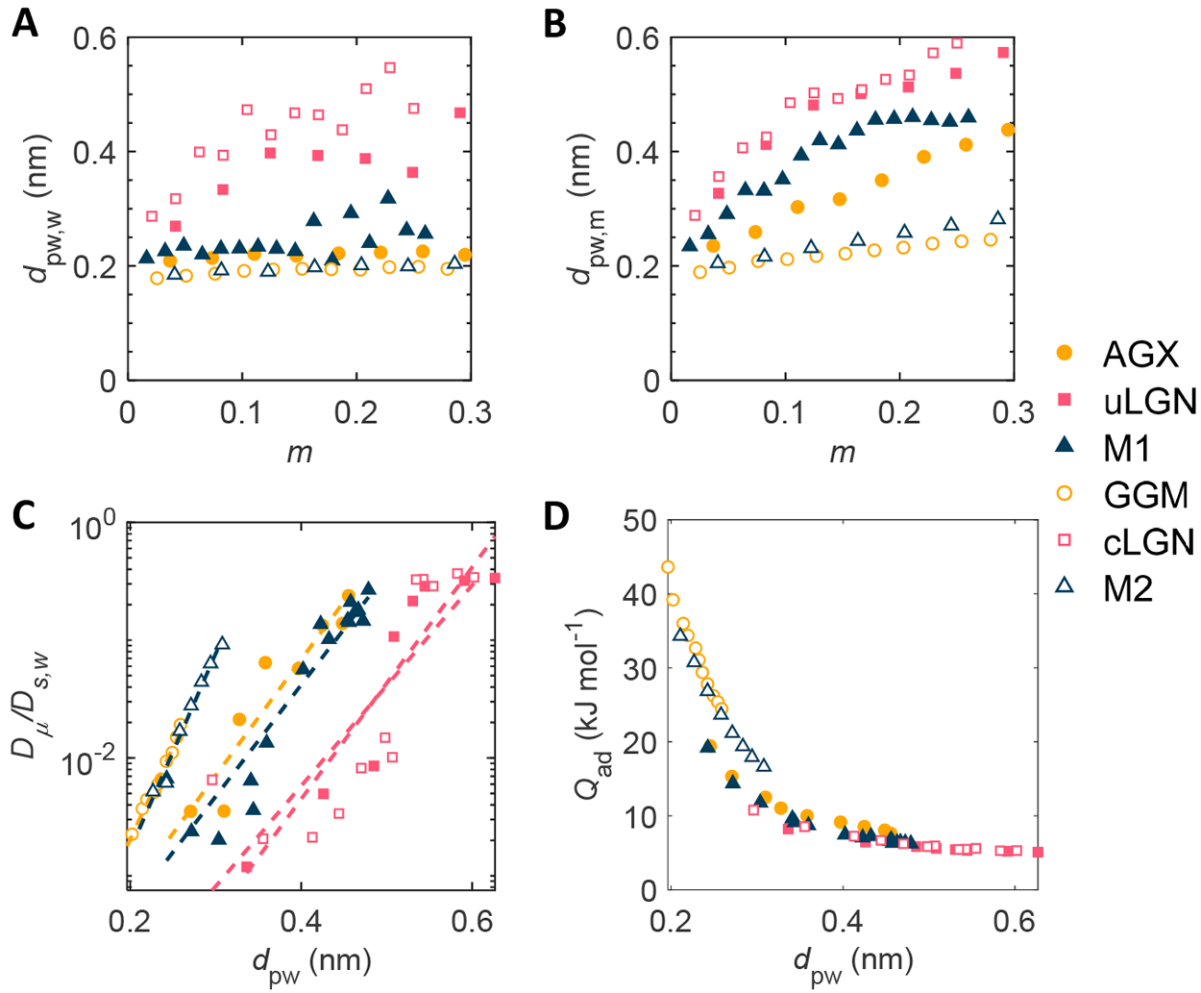
Polymer–water distance in relation with diffusion coefficient and adsorption heat. (A and B) Average polymer–water distance of waiting and moving state, respectively. The *d*_pw,m_ approximates *d*_pw_ because most water molecules are in a moving state, τ_m_*v* ∼ 1. (C) Normalized diffusivity in relation to polymer–water distance *d*_pw_. (D) Adsorption heat is strongly correlated with polymer–water distance.

The mobility status is found to correlate with the polymer–water distance *d*_pw_, defined as the average distance between the oxygen atom of water and its nearest polymer atoms. The more mobile water molecules locate further away from the polymer, *d*_pw,m_ > *d*_pw,w_, shown in Figure 3. Factually, *d*_pw_ can characterize the dispersity of water because it is related to the ratio of the number of water at the surface of the cluster to the total number of water of the cluster, a common measure of dispersity. Based on our results, water in hemicelluloses disperses better than in lignins, a behavior that agrees with previous reports.^22^

### Microscopic Diffusion Coefficients and Tortuosity

In the following, we analyze the behavior of the ratio *D*_μ_/*D*_s,w_, referred to as the normalized diffusion coefficient, which is the microscopic diffusion coefficient normalized by the bulk self-diffusion coefficient. We note that the reciprocal of *D*_μ_/*D*_s,w_ is often referred to as tortuosity, ξ = *D*_s,w_/*D*_μ_, interpreted as the degree of “detour” of the path taken by a diffusing water molecule caused by the tortuous pore space. The normalized diffusion coefficient ξ^–1^ is usually less than 1, ξ^–1^ < 1, because the diffusion coefficient in porous media is generally less than the bulk self-diffusion coefficient of liquid water under the same thermodynamic conditions.

**Figure 4:**
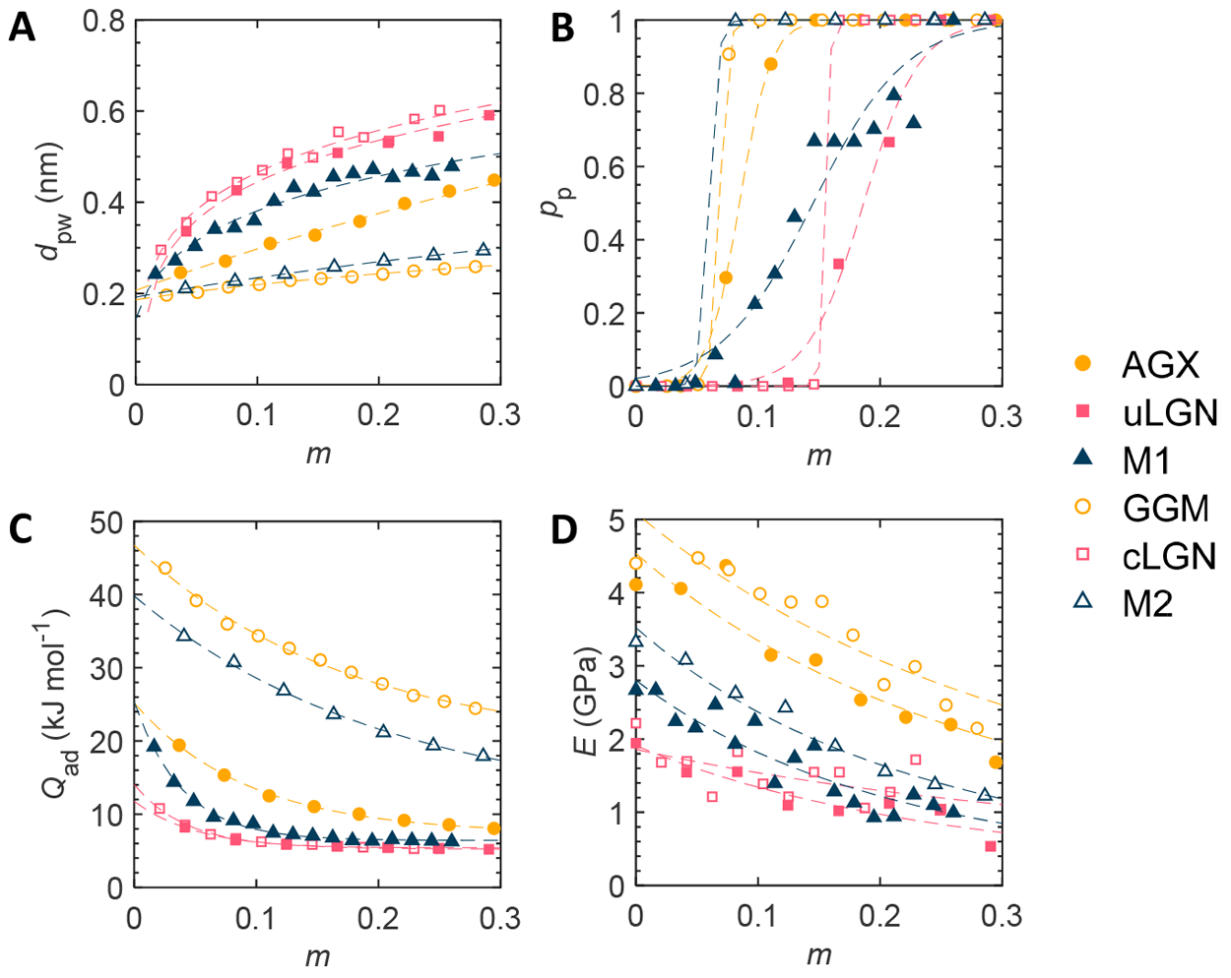
Material properties as a function of moisture content *m*. (A) Average polymer–water distance *d*_pw_. Trend line using *d*_pw_ = (*m* – *a*)^*b*^ + *c*. With increasing moisture content, the average position of water molecules moves away from the polymer surface as expected. (B) Percolation probability *p*_p_. Trend line using *p*_p_ = 1/(exp(−a*m + *b*) + 1). (C) Heat of adsorption *Q*_ad_. Trend line using *Q*_ad_ = a* exp(−b*m) + c. (D) Young’s modulus *E*. Trend line using *E* = a*(1 + *m*)^*b*^.

We relate diffusion to sorption and deformability, which are quantitatively represented by adsorption heat *Q*_ad_ and Young's modulus *E*, respectively. The material properties as a function of moisture content are shown in Figure 4. Details of measurement methods are described in Supplementary Note S4. The normalized diffusion coefficient or tortuosity is an exponential function of adsorption heat and elastic moduli. The justification is discussed below.

**Figure 5:**
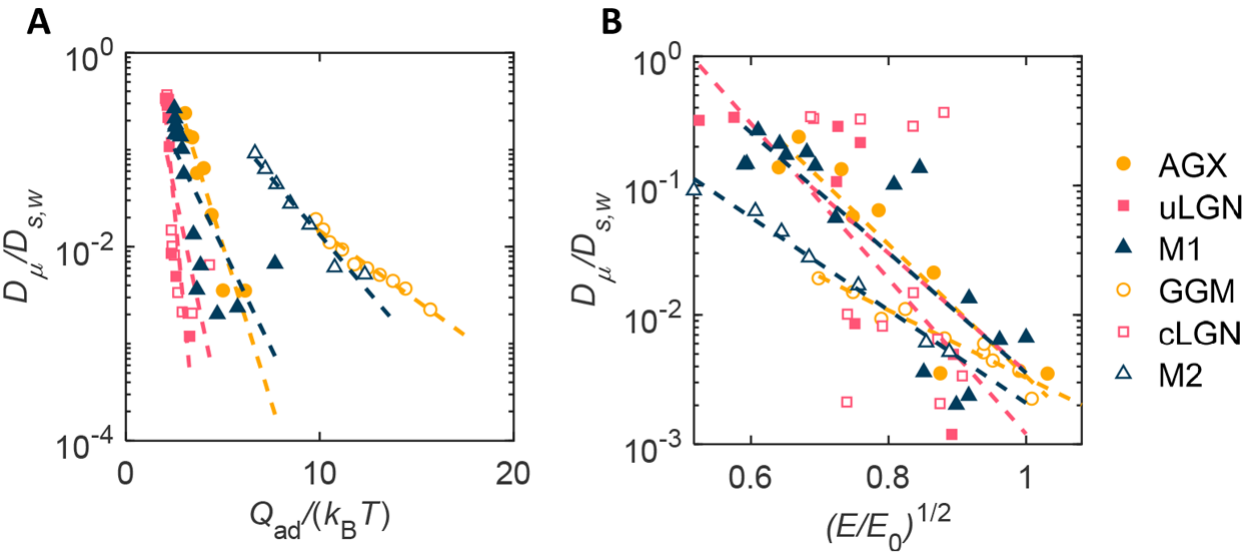
Normalized diffusion coefficient in relation to material properties. (A) Heat of adsorption *Q*_ad_/*k*_B_*T* and (B) Young’s modulus (*E*/*E*_0_)^1/2^ in a semilog plot.

In the activation theory, the diffusion coefficient scales with the activation energy *E*_a_, *D* ∼ exp(−*E*_a_/*k*_B_*T*). One can invoke a linear relationship between activation energy *E*_a_ of diffusion and heat of adsorption, *E*_a_ ∼ *αQ*_ad_ (with α on the order but larger than 1; in other words, the energy barrier is larger than the energy difference between two states).^30^ Therefore, taking the limit α = 1, an exponential relation between normalized diffusion coefficient can be employed, ξ^–1^ ∼ exp(−*Q*_ad_/*k*_B_*T*), where *k*_B_ and *T* denote the Boltzmann constant and temperature, respectively. This relation is shown to be valid, as seen in Figure 5a. It should be noted that in this study we take liquid water as the reference state.

Yang and Chun derived the relation between diffusion coefficient and polymer persistence length ln *D* ∼ *l*_p_^1/2^.^31^ The persistence length of a worm-like chain is related to Young’s modulus *E*, cross-section area, and moment of inertia *M*, following the relation *l*_p_ = *EM*/*k*_B_*T*.^32^ Therefore, the normalized diffusion coefficient is also assumed to scale exponentially with Young’s modulus, ξ^–1^ ∼ exp[(*E*/*E*_0_)^1/2^], where *E*_0_ is simply assumed as 1 GPa to make the term between brackets dimensionless. Figure 5b and Figure S4 show the validity of the proposed exponential scaling.

The prediction of diffusion coefficient based on heat of adsorption and elastic moduli works relatively well, as shown in Supplementary Note S5. However, for some systems, *e.g*., cLGN, there exists a surge of diffusion coefficients around *m* ∼ 0.15, which is related to the occurrence of percolation. The prediction above, which relies on two terms (heat of adsorption and elastic moduli), fails to include such a feature. It is thus necessary to introduce percolation into the model.

In this study, percolation is quantified by the percolation probability *p*_p_ defined as the time interval during which percolation is occurring divided by the total simulation time, *i.e.*, *p*_p_ = ∫ δ_p_(*t*)d*t*/∫ d*t*, where δ_p_(*t*_*i*_) equals 1 or 0 whether or not the system percolates at time *t*_*i*_, correspondingly (details in Supplementary Note S4). The scaling between the normalized diffusion coefficient and percolation probability *p*_p_ is not a simple function. In analogy with heat of adsorption and modulus, we propose an exponential scaling, ξ^–1^ ∼ exp(*p*_p_) (it will be shown afterward that this mathematical form provides a reasonable description of the obtained results). In essence, percolation is a special type of structural feature that is not represented by heat of adsorption and elastic modulus, which supports the inclusion of percolation as a separate factor. The percolation probability is system size dependent. The size dependency is not considered as a critical issue here because percolation probability will be paired with an adjustable parameter that incorporates in an effective manner all unspecified effects including the size effect, shown below.

In the spirit of tortuosity factorization,^33^ the above-mentioned factors are multiplied in the sorption–deformation–percolation (SDP) model:
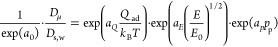
where *a*_*i*_ (*i* = 0, *Q*, *E*, or *p*) is weight. We can also write
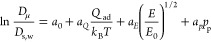


**Figure 6:**
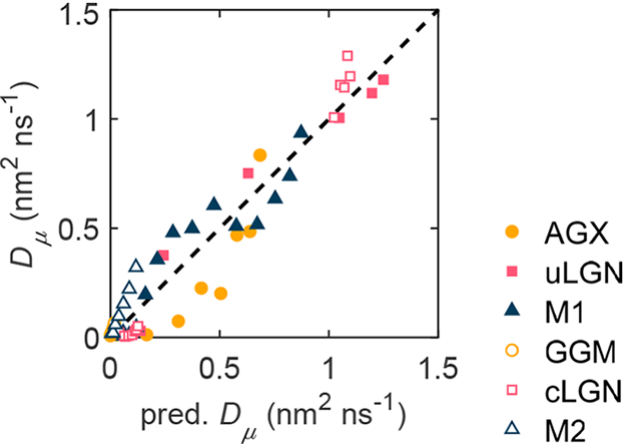
Measured and predicted diffusion coefficient *D*_*μ*_.

The SDP model well describes the measurements as shown in Figure 6 and Figure S3 (systems are separated into individual plots). A sensitivity analysis is carried out by omitting an arbitrary number of factors. The prediction is poor for any model with fewer factors.

The weights are *a*_0_ = −0.572, *a*_*Q*_ = −3.051, *a*_*E*_ = −1.243, and *a*_*p*_ = 0.466. The weights *a*_*i*_ in the SDP model are dimensionless numbers, which are the first derivative of ln ξ^–1^ with respect to the corresponding property *x*_*i*_, *a*_*i*_ = ∂ ln ξ^–1^/∂*x*_*i*_. The *a*_0_ is introduced to manifest all the influencing factors other than sorption, deformation, and percolation. When *a*_*Q*_ is zero, the SDP model reduces to the description of a nonsorptive material. Unlike most previous diffusion studies, which shun complexity by assuming negligible host–guest interaction and pore length scale being much larger than the diffusant size, here the SDP model tackles the challenge of diffusion in dense, sorptive, and deformable polymer materials.

The SDP model can be verified by experiments. The diffusion coefficient can be measured via nuclear magnetic resonance (NMR). The waiting and moving statistics are accessible via experimental methods such as differential scanning calorimetry (DSC) and NMR.^34^ In fact, our simulation shows that water in most systems is mainly in the moving state, except for the strongly adsorbing material, GGM, whose heat of adsorption reaches ∼43 kJ mol^–1^. It is noted that the latent heat of water is not included, as we use liquid water as the reference state (details in Supplementary Note S4). The proposed material parameters of tortuosity are also experimentally accessible: heat of adsorption *Q*_ad_ and Young’s modulus *E* can be measured via calorimetry and mechanical tests, respectively; the probability of percolation can be estimated by, for example, conductivity or capacitance. Preliminary validation of the SDP model based on literature data is included in Supplementary Note S6, where acceptable agreement between the predicted and measured diffusion coefficient is shown.

### Discussion

Stop-and-go is a simple picture of water motion in sorptive systems. There are other modes of (semi)residence such as surface diffusion where water is confined to a sorptive surface but is free to travel within the vicinity of the surface.^35^ In a highly confined porous system, it is difficult to distinguish residence modes. In fact, defining the surface, at the nanoscale, can be a highly nontrivial task.

While this study focuses on water diffusion, we believe that our framework can be extended to other diffusants, such as hydrated ions and methane, as long as the molecular size approximates the size of the free volume or pore length scale. Such a scenario has rarely been covered by previous studies, however, is of significant academic and industrial relevance. For instance, most drugs have molecular sizes around 0.5–5 nm, which are comparable to the pore length scale of the drug delivery systems. Better control of drug release can be achieved by the rational design of the structure of the host porous media under the guidance of our model.

The rigorous applicability of the SDP model to other polymer systems remains to be verified by comprehensive studies of polymers with different chemistry, structure, and conditions. The current study motivates further studies by offering a concise physical picture of diffusion.

## Conclusion

This study offers a unifying picture of water diffusion within dense disordered deformable porous media and moreover describes the diffusion coefficient via a complete set of physically well-defined material properties, *i*.*e*., heat of adsorption *Q*_ad_, Young’s modulus *E*, and probability of percolation *p*_p_. We resort to MD simulations where the trajectories of individual water molecules manifest the intermittent dynamics, which is caused by strong sorption interactions and highly tortuous pore space. The microscopic diffusion coefficient *D*_μ_ should be extracted from the measured diffusion coefficient *D* by disregarding the waiting phases using the factor τ_m_*v*, the fraction of moving water derived from single water molecule statistics. The microscopic diffusion coefficient, equivalently tortuosity ξ, is then predicted via three characteristic parameters of the porous media, *i.e.*, *Q*_ad_, *E*, and *p*_p_, each representing a fundamental physical aspect of the porous system, *i*.*e*., sorption, deformation, and percolating structure. The SDP model, therefore, provides guidelines for the comparison of diffusion in different polymers. The model may guide the design of materials to achieve the desired diffusion property.

## Materials and Methods

Following our previous work,^22^ six different polymer systems are built. AGX consists of three types of monomers, *i*.*e*., 67% xylose, 20% glucuronoacid-xylose, and 13% arabinoxylose, which are randomly polymerized.^28^ GGM consists of two types of monomers, *i*.*e*., 25% glucose and 75% mannose branched with galactose side groups (∼8%).^36^ The uLGN is a linear homopolymer of coniferyl units.^37^ The cLGN is a randomly branched polymer of coniferyl units with 60% β-O-4 linkage and 40% 5–5′ linkage.^38^ In addition to the single-component systems, AGX and uLGN are randomly mixed, forming matrix 1 (M1) with a mass ratio of 1:2. Similarly GGM and cLGN randomly form M2 with a mass ratio of 7:4.^39−42^ The detailed dimensions and densities of polymer systems are listed in Table 1.

**Table 1:** Dimension Details of Polymeric Systems Studied

	component	# monomers per chain	# chains	volume (nm^3^)	density(g cm^–3^)
hemicellulose	AGX	100	5	116.2	1.30
	GGM	100	4	91.1	1.24
lignin	uLGN	100	5	123.0	1.33
	cLGN	1–43	136	123.4	1.30
matrix 1	AGX in M1	50	4	71.2	1.28
	uLGN in M1	50	4		
matrix 2	GGM in M2	100	3	116.4	1.24
	cLGN in M2	1–43	19		

A typical simulation system has a lateral size of ∼5 nm, containing ∼10,000 atoms. The MD simulations are carried out using the GROMACS 5.0 package^43^ and GROMOS 53a6 united-atom force field^44^ in an isobaric–isothermal ensemble realized by a velocity rescaling thermostat^45^ and a Berendsen barostat.^46^ The simulation time of each system is 100–1000 ns depending on the time needed to reach the Fickian diffusion regime, *i*.*e*., where the MSD scales linearly with time. Sorption is mimicked by the random insertion method. Following each successful insertion, energy minimization and 100 ps relaxation are carried out.^22^ More details are in Supplementary Note S2.

### Material Properties

Percolation probability *p*_p_ is defined as the time interval during which percolation is occurring divided by the total simulation time, *i.e.*, *p*_p_ = ∫δ_p_(*t*) d*t*/∫ d*t*, where δ*p*(*t*_*i*_) equals 1 or 0 when the system percolates or not at time *t*_*i*_, correspondingly. Integral heat of adsorption is defined as *Q*_ad_ = *H*_p_ + *H*_wv_ – *H*_c_)/*n*_water_ – *H*_latent_, where *H*_p_, *H*_wv_, *H*_c_, *n*_water_, and *H*_latent_ are the enthalpies of dry polymer, water vapor, polymer–water mixture, the amount of water in moles, and latent heat of water, respectively. *H*_p_ and *H*_c_ are calculated with the definition of enthalpy *H* = *U* + *PV*, where *U*, *P*, and *V* are directly measurable from MD. *H*_wv_ is taken as 4*n*_water_*RT*.^28^*H*_latent_ takes the value of 40.68 kJ mol^–1^.^47^ The elastic constant, *i*.*e*., Young’s modulus, is determined from the linear regime of stress–strain curves of uniaxial tensile tests.^22^ Stepwise strains are applied with each step straining around 0.01% of the initial dimension until a total strain of ∼1%. Every strain step is followed by a relaxation run of 100 ps, where the tension strain is maintained while the transverse directions are subjected to stress-free relaxation.

## Supporting Information Available

Definitions of diffusion coefficients; chemical structures and molecular models of polymers; mean square displacement curves and diffusivity; percolation, adsorption heat, elastic modulus, and polymer–water distance; normalized diffusivity predicted by adsorption heat and elastic moduli; validation of the sorption–deformation–percolation model; others (PDF)
