# Supplementary material for: Sorption–Deformation–Percolation Model for Diffusion in Nanoporous Media
Source: ACS Nano. 2023 Feb 27;17(5):4507–14. doi: 10.1021/acsnano.2c10384 (PMC10018761; doi:10.1021/acsnano.2c10384)
Supplement: Supplementary file 1 — nn2c10384_si_001.pdf [file nn2c10384_si_001.pdf]

Supplementary Materials for

Sorption-deformation-percolation model for diffusion in nanoporous media

Chi Zhang[†*], *Ali Shomali*[†], *Benoit Coasne*[‡], *Dominique Derome*[§], *Jan Carmeliet*[†]

[†]Chair of Building Physics, Department of Mechanical and Process Engineering, ETH Zurich, Raemistrasse 101, 8092 Zurich, Switzerland.

[‡] Université Grenoble Alpes, CNRS, LIPhy, 38000 Grenoble, France

[§]Department of Civil and Building Engineering, Université de Sherbrooke, Sherbrooke J1K 2R1, Québec, Canada

* Email: outlook.zhangchi@gmail.com

Note S1. Definitions of diffusion coefficients

Fick's first law relates the molecular flux j to concentration gradient ∇c and a proportionality $D_t(c)$ which is called transport diffusion coefficient (also collective, chemical or center of mass diffusivity). Nonetheless, a more rigorous relation should replace the concentration gradient with the chemical potential gradient. The transport diffusion coefficient or diffusivity $D_t(c)$ equals the corrected diffusion coefficient or diffusivity $D_c(c)$ multiplied with a thermodynamic correction factor. The correction factor describes the relation between activity and concentration, *i.e.* the information provided by the sorption isotherm.

$$D_t(c) = D_c(c)\frac{\mathrm{dln}p}{\mathrm{dln}c} \tag{1}$$

This multiplication equation is reminiscent of the so-called solution-diffusion model where permeability is derived from the product of partition, namely the sorption affinity and diffusivity [1].

The corrected diffusivity can be calculated as:

$$D_c(c) = \frac{1}{2dN}\lim_{t\to\infty}\frac{d}{dt}\left\langle\left(\sum_{l=1}^{N}[\boldsymbol{r}_l(\tau)-\boldsymbol{r}_l(0)]\right)^2\right\rangle \tag{2}$$

Self-diffusivity is equivalent to tracer diffusivity [2] and can be calculated via Einstein's equation:

$$D_s(c) = \frac{1}{2dN}\lim_{t\to\infty}\frac{1}{t}\left\langle\sum_{l=1}^{N}(\boldsymbol{r}_l(\tau)-\boldsymbol{r}_l(0))^2\right\rangle \tag{3}$$

where d, N, $\boldsymbol{r}_l$ and τ are the dimensionality of the system, total number of the particles, position of particle l and time interval respectively.

There seems to be a confusion between self-diffusivity and corrected diffusivity in literature. For example, IUPAC refers to the corrected diffusivity in eq. (2) as the self-diffusivity [3]. It is necessary to clarify that, as shown by the equations above, that the corrected diffusivity tracks the collective motion of the diffusing species, whereas self-diffusivity tracks the average motion of individual particles. The transport, corrected and self-diffusivities strictly coincide only for concentration approaching zero.

Fick's first law relates molecular flux j to concentration gradient ∇c and transport/collective diffusivity $D_t(c)$ which approximates self-diffusivity when concentration approaches zero. Therefore, self-diffusivity can also provide fundamental insights in transport processes driven by gradient [4].

Note S2. Chemical structures and molecular models of polymers

AGX consists of three types of monomers, *i.e.*, 67% xylose, 20% glucuronoacid-xylose, and 13% arabinoxylose, which are randomly polymerized [5]. GGM consists of two types of monomers, *i.e.* 25% glucose and 75% mannose branched with galactose side groups (~8 wt. %) [6]. The uLGN is a linear homopolymer of coniferyl units [7]. The cLGN is a randomly branched polymer of coniferyl units with 60% of β-O-4 linkage and 40% of 5-5' linkage [8]. In addition to the single-component systems, AGX and uLGN are randomly mixed forming M1 with a mass ratio of 1:2. Similarly GGM and cLGN randomly form M2 with a mass ratio of 7:4 [9–12]. For more details on models, we refer to our previous work [13].

A typical simulation system is of lateral size of ~5 nm, containing ~10,000 atoms. The MD simulations are carried out using GROMACS 5.0 package [14] and GROMOS 53a6

united-atom force field [15] in isobaric-isothermal ensemble realized by velocity rescaling thermostat [16] and Berendsen barostat [17] for >100 ns. The systems are equilibrated in the isobaric-isothermal ensemble at 300 K and 0 bar using the velocity rescaling thermostat [16] and Berendsen barostat [17]. Time step is set to 1 fs. All hydrogen-related bonds are constrained by LINCS algorithm [18]. The van der Waals interaction is cut-off at 1 nm distance. The long-range electrostatic interactions are computed using particle-mesh Ewald summation. The simulation time of each system is 100-1000 ns depending on the time needed to reach the Fickian diffusion regime.

Hydration is mimicked with the random insertion method. Single point charge (SPC) models of water are introduced into the dry polymer system via random insertion [13]. Following each successful insertion, energy minimization and 100 ps relaxation are carried out.

Note S3. Mean square displacement curves and diffusivity

Mean square displacement (MSD), *i.e.* $(\boldsymbol{r}(t) - \boldsymbol{r}(t=0))^2$, quantifies how far water molecules have traveled versus time in reference to their initial locations at initial time $t = 0$, $\boldsymbol{r}(t=0)$. The good linearity of the log-log plots of MSD vs τ evidences the diffusion to be within the Fickian regime. Nevertheless, it is noted that, for some systems at low moisture content, *e.g.* AGX at $m \sim 0.037$, the diffusion never reached the Fickian regime even after 1000 ns of simulation. The diffusion coefficients of such cases, therefore, are not available. But such cases should not affect the main conclusions of the current study.

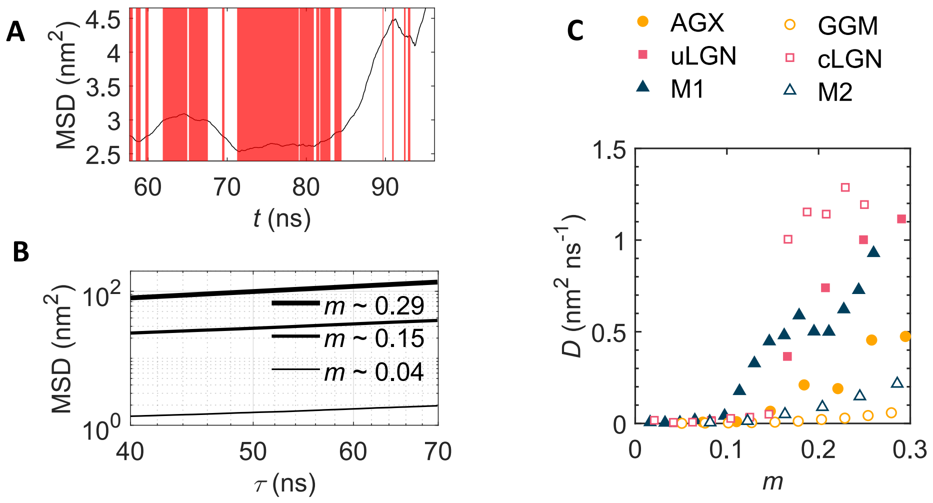

Figure S1. Mean square displacement and diffusion coefficient. (**A**) Sample MSD curve of one water molecule. Red areas denote waiting state. (**B**) Sample MSD curves averaged over all water molecules on a log-log plot of AGX at different moisture contents. (**C**) Diffusivity measured versus moisture content for different polymers.

Note S4. Percolation, adsorption heat, elastic modulus and polymer-water distance

Percolation is defined as the occurrence of one large water cluster spanning from one side to the opposite side of the simulation box. A pair of water molecules are considered to be in the same water cluster when their oxygen-oxygen distance is less than 0.6 nm, a value corresponding to the second minimum of the radial distribution function of bulk water. Percolation probability p_{p} is defined as the time interval during which percolation is occurring divided by the total simulation time, *i.e.* $p_{\mathrm{p}} = \int \delta_{\mathrm{p}}(t)dt / \int dt$, where $\delta_{\mathrm{p}}(t_i)$ equals 1 or 0 when the system percolates or not at time t_i, correspondingly. Polymer-water distance and percolation probability are generally system size dependent. However, this study focuses on dense polymer networks with nanoscale pores, which minimizes such possible size effect.

Integral heat of adsorption is employed here to quantify the total heat released by water vapor being adsorbed:

$$Q_{\mathrm{ad}} = (H_{\mathrm{p}} + H_{\mathrm{wv}} - H_{\mathrm{c}})/n_{\mathrm{water}} - H_{\mathrm{latent}} \tag{4}$$

where H_{p}, H_{wv}, H_{c}, n_{water} and H_{latent} are the enthalpies of dry polymer, water vapor, polymer-water mixture, the amount of water in mole and latent heat of water, respectively. H_{p} and H_{c} are calculated with the definition of enthalpy $H = U + PV$, where U, P and V are directly measurable from MD. H_{wv} is taken as $4n_{\mathrm{water}}RT$ [5]. H_{latent} takes the value of 40.68 kJ mol^{-1} [19].

The elastic constant, *i.e.* Young's modulus, is determined from the linear regime of stress-strain curves of uniaxial tensile tests [13]. Stepwise strains are applied with each step straining around 0.01% of the initial dimension until a total strain of ~1%. Every strain step is followed by a relaxation run of 100 ps, where the tension strain is maintained while the transverse directions are subjected to stress-free relaxation.

Note S5. Normalized diffusivity predicted by adsorption heat and elastic moduli

Without the consideration of percolation, the normalized diffusivity is predicted by adsorption heat and Young's modulus using the follow equation:

$$\frac{1}{\exp\,(a_0)} \cdot \frac{D_\mu}{D_{s,w}} = \exp\left(a_Q \frac{Q_{\mathrm{ad}}}{k_{\mathrm{B}}T}\right) \cdot \exp\left(a_E \left(\frac{E}{E_0}\right)^{\frac{1}{2}}\right) \tag{5}$$

or equivalently, $\ln \frac{D_\mu}{D_{s,w}} = a_0 + a_Q \frac{Q_{\mathrm{ad}}}{k_{\mathrm{B}}T} + a_E \left(\frac{E}{E_0}\right)^{\frac{1}{2}}$

where a_i ($i = 0$, Q or E), k_{B}, T, E_0 are weights, Boltzmann constant, temperature and normalizing value of 1 GPa, respectively. The weights are $a_0 = 0.370$, $a_Q = -2.93$, $a_E = -0.198$. The comparison between the measurement and model prediction is shown in Figure S2.

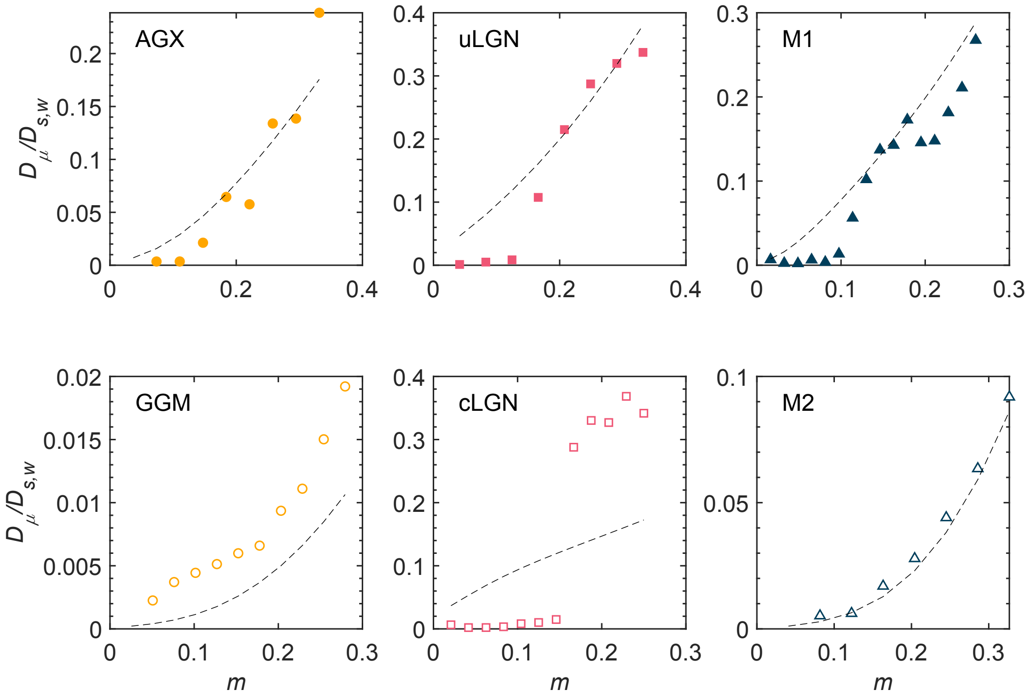

Figure S2. Measurement (data points) and model prediction (dashed lines) of normalized diffusivity $\xi^{-1} = D_\mu/D_{s,w}$ vs. moisture content m. Percolation is not included in the model.

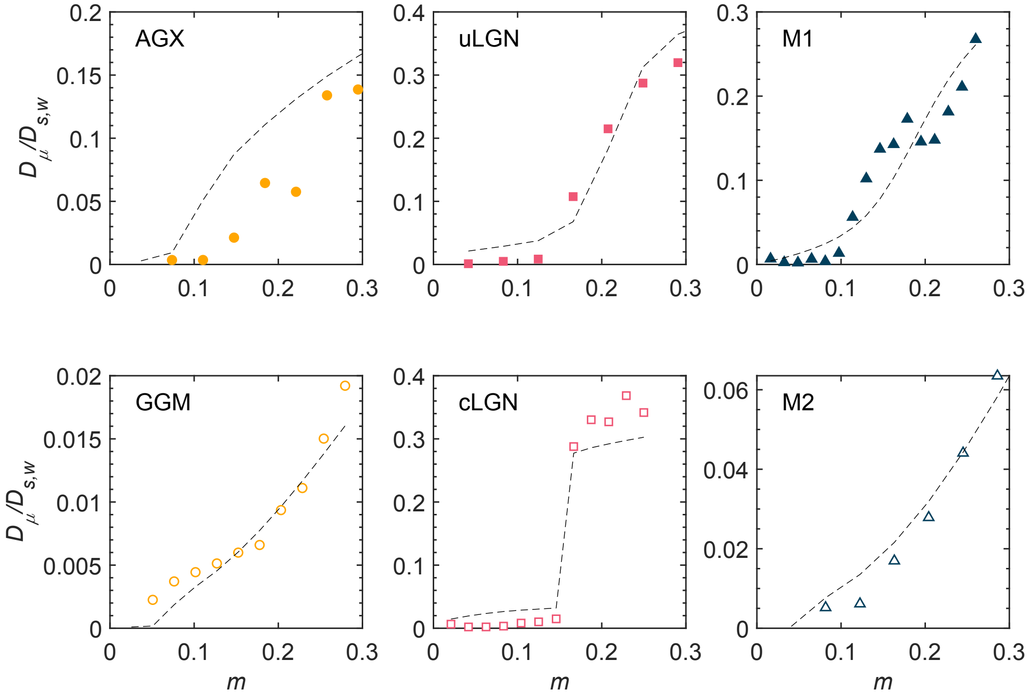

Figure S3. Measurement (data points) and model prediction (dashed lines) of normalized diffusivity $\xi^{-1} = D_{\mu}/D_{s,w}$ vs. moisture content *m*. Percolation is included in the model.

Note S6. Validation of the sorption-deformation-percolation (SDP) model

The SDP model is validated via literature data, including amorphous cellulose, epoxy and polyhydroxybutyrate, shown in Table S1. The percolation probability is rarely reported in literature. Therefore, estimation is made based on the hydration state: 1 for highly hydrated system and 0 for low hydration. The prediction power is estimated to be satisfactory, especially considering the simplicity of the SDP model. A more rigorous validation would allow comprehensive investigation of diverse polymers. While this is an interesting task, we consider this opportunity as a possible topic of future study.

Table S1 Comparison of model prediction and literature reports.

**material**	**heat of adsorption**	**Young's modulus**	**percolation probability**	**measured diffusion coefficient**	**SDP model prediction**
	**kJ mol^{-1}**	**Gpa**		**nm^2 ns^{-1}**	**nm^2 ns^{-1}**
amorphous cellulose	5.4 [20]	3.5 [20]	1	~0.2 [20]	0.1967
epoxy	18.83 [21]	3.2 [22]	1	3E-04 [22]	2.6E-3
polyhydroxy butyrate	10-90 [23]	1.2 [24]	0	~1.9E-3 [25]	1.80E-3

Note S7. Others

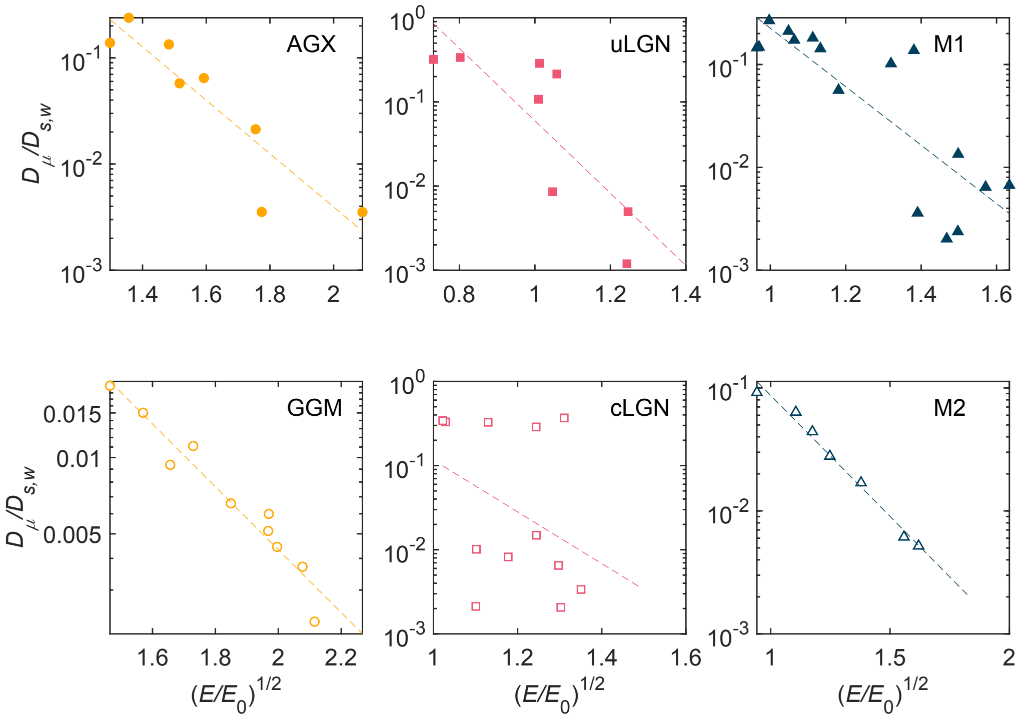

Figure S4. Normalized diffusion coefficient in relation to Young's modulus $(E/E_0)^{1/2}$ in a semi-log plot.

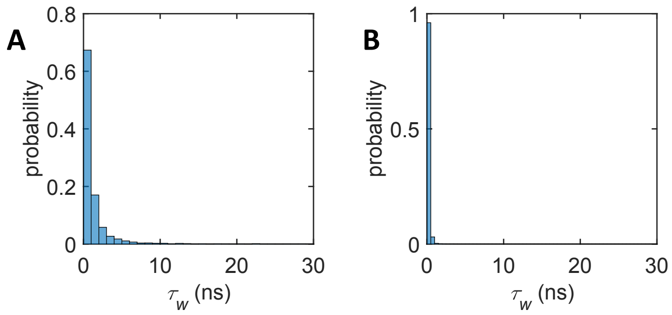

Figure S5. Sample histograms of waiting time τ_w of (A) AGX at m ~ 0.03, (B) AGX at m ~ 0.22.

References

(1) Kanduč, M.; Kim, W. K.; Roa, R.; Dzubiella, J. How the Shape and Chemistry of Molecular Penetrants Control Responsive Hydrogel Permeability. *ACS Nano* **2021**, *15* (1), 614–624. https://doi.org/10.1021/acsnano.0c06319.

(2) Kärger, J.; Ruthven, D. M. Diffusion in Nanoporous Materials: Fundamental Principles, Insights and Challenges. *New J. Chem.* **2016**, *40* (5), 4027–4048. https://doi.org/10.1039/c5nj02836a.

(3) McNaught, A. D.; Wilkinson, A. *The IUPAC Compendium of Chemical Terminology*; Gold, V., Ed.; International Union of Pure and Applied Chemistry (IUPAC): Research Triangle Park, NC, 2019. https://doi.org/10.1351/goldbook.

(4) Falk, K.; Coasne, B.; Pellenq, R.; Ulm, F. J.; Bocquet, L. Subcontinuum Mass Transport of Condensed Hydrocarbons in Nanoporous Media. *Nat. Commun.* **2015**, *6* (1), 6949. https://doi.org/10.1038/ncomms7949.

(5) Zhang, C.; Coasne, B.; Guyer, R.; Derome, D.; Carmeliet, J. Moisture-Induced Crossover in the Thermodynamic and Mechanical Response of Hydrophilic Biopolymer. *Cellulose* **2020**, *27* (1), 89–99. https://doi.org/10.1007/s10570-019-02808-z.

(6) Kulasinski, K.; Guyer, R.; Keten, S.; Derome, D.; Carmeliet, J. Impact of Moisture Adsorption on Structure and Physical Properties of Amorphous Biopolymers. *Macromolecules* **2015**, *48* (8), 2793–2800. https://doi.org/10.1021/acs.macromol.5b00248.

(7) Salmén, L.; Burgert, I. Cell Wall Features with Regard to Mechanical Performance. A Review COST Action E35 2004–2008: Wood Machining – Micromechanics and Fracture. *Holzforschung* **2009**, *63* (2), 121–129. https://doi.org/10.1515/HF.2009.011.

(8) Åkerholm, M.; Salmén, L. Interactions between Wood Polymers Studied by Dynamic FT-IR Spectroscopy. *Polymer (Guildf).* **2001**, *42* (3), 963–969. https://doi.org/10.1016/S0032-3861(00)00434-1.

(9) Pettersen, R. C. The Chemical Composition of Wood; Rowell, R., Ed.; Advances in Chemistry; American Chemical Society: Washington, DC, 1984; Vol. 207, pp 57–126. https://doi.org/10.1021/ba-1984-0207.ch002.

(10) Dinwoodie, J. M. *Timber: Its Nature and Behaviour*; Routledge, London, 2000.

(11) Scheller, H. V.; Ulvskov, P. Hemicelluloses. *Annu. Rev. Plant Biol.* **2010**, *61* (1), 263–289. https://doi.org/10.1146/annurev-arplant-042809-112315.

(12) Hon, D. N.-S.; Shiraishi, N. *Wood and Cellulosic Chemistry. Second Edition, Revised and Expanded*; Cambridge University Press, 2013; Vol. 53. https://doi.org/10.1017/CBO9781107415324.004.

(13) Zhang, C.; Chen, M.; Keten, S.; Coasne, B.; Derome, D.; Carmeliet, J. Hygromechanical Mechanisms of Wood Cell Wall Revealed by Molecular Modeling and Mixture Rule Analysis. *Sci. Adv.* **2021**, *7* (37). https://doi.org/10.1126/sciadv.abi8919.

(14) Abraham, M. J.; Murtola, T.; Schulz, R.; Páll, S.; Smith, J. C.; Hess, B.; Lindah, E. Gromacs: High Performance Molecular Simulations through Multi-Level Parallelism from Laptops to Supercomputers. *SoftwareX* **2015**, *1–2*, 19–25. https://doi.org/10.1016/j.softx.2015.06.001.

(15) Oostenbrink, C.; Villa, A.; Mark, A. E.; Van Gunsteren, W. F. A Biomolecular

Force Field Based on the Free Enthalpy of Hydration and Solvation: The GROMOS Force-Field Parameter Sets 53A5 and 53A6. *J. Comput. Chem.* **2004**, *25* (13), 1656–1676. https://doi.org/10.1002/jcc.20090.

(16) Bussi, G.; Donadio, D.; Parrinello, M. Canonical Sampling through Velocity Rescaling. *J. Chem. Phys.* **2007**, *126* (1), 014101. https://doi.org/10.1063/1.2408420.

(17) Berendsen, H. J. C.; Postma, J. P. M.; van Gunsteren, W. F.; DiNola, A.; Haak, J. R.; van Postma, J. P. M.; van Gunsteren, W. F.; DiNola, A.; Haak, J. R. Molecular Dynamics with Coupling to an External Bath. *J. Chem. Phys.* **1984**, *81* (8), 3684–3690. https://doi.org/10.1063/1.448118.

(18) Hess, B.; Bekker, H.; Berendsen, H. J. C.; Fraaije, J. G. E. M. LINCS: A Linear Constraint Solver for Molecular Simulations. *J. Comput. Chem.* **1997**, *18* (12), 1463–1472. https://doi.org/10.1002/(SICI)1096-987X(199709)18:12<1463::AID-JCC4>3.0.CO;2-H.

(19) Murphy, D. M.; Koop, T. Review of the Vapour Pressures of Ice and Supercooled Water for Atmospheric Applications. *Q. J. R. Meteorol. Soc.* **2005**, *131* (608), 1539–1565. https://doi.org/10.1256/qj.04.94.

(20) Kulasinski, K. Physical and Mechanical Aspects of Moisture Adsorption in Wood Biopolymers Investigated with Atomistic Simulations, 2015. https://doi.org/10.3929/ethz-a-010564673.

(21) Yang, C.; Han, Q.; Wang, A.; Han, W.; Sun, L.; Yang, L. Study on Diffusion Mechanism and Failure Behavior of Epoxy Coatings Focusing on Synergistic Effect of Temperature and Water Molecules. *Des. Monomers Polym.* **2021**, *24* (1), 73–88. https://doi.org/10.1080/15685551.2021.1904581.

(22) Vertuccio, L.; Sorrentino, A.; Guadagno, L.; Bugatti, V.; Raimondo, M.; Naddeo, C.; Vittoria, V. Behavior of Epoxy Composite Resins in Environments at High Moisture Content. *J. Polym. Res.* **2013**, *20* (6), 178. https://doi.org/10.1007/s10965-013-0178-5.

(23) Rosa, G. S. da; Godoi, F. C. de; Marsaioli, A.; Rocha, C. dos S. Equilibrium Isotherms of a Biodegradable Polymer Polyhydroxybutyrate (PHB): Heat of Desorption. *Chem. Eng. Trans.* **2011**, *24*, 661. https://doi.org/10.3303/CET1124111.

(24) Marinho, V. A. D.; Carvalho, L. H.; Canedo, E. L. Effect of Water Absorption on the Mechanical Properties of Poly(3-Hydroxybutyrate)/Vegetable Fiber Composites. *AIP Conf. Proc.* **2015**, *1664*. https://doi.org/10.1063/1.4918421.

(25) Iordanskii, A. L.; Razumovskii, L. P.; Krivandin, A. V.; Lebedeva, T. L. Diffusion and Sorption of Water in Moderately Hydrophilic Polymers: From Segmented Polyetherurethanes to Poly-3-Hydroxybutyrate. *Desalination* **1996**, *104* (1–2), 27–35. https://doi.org/10.1016/0011-9164(96)00023-9.
